# Global Availability of Cancer Registry Data

**DOI:** 10.1200/JGO.18.00116

**Published:** 2018-07-26

**Authors:** Asif H. Siddiqui, Syed Nabeel Zafar

**Affiliations:** **Asif H. Siddiqui**, Aga Khan University, Karachi, Pakistan; and **Syed Nabeel Zafar**, University of Maryland Medical Center, Baltimore MD.

## TO THE EDITOR:

The availability of cancer registries has significantly enhanced cancer research, especially that related to cancer epidemiology, survival, interventions, and outcomes.^1^ However, by using publicly available sources, we aimed to map the availability and extent of cancer registry data in each country. We also aimed to test the association of registry data with metadata such as country income and national cancer-related policies.

Data from 190 countries were collected from the WHO country cancer profiles.^2^ We sought data on the presence of an operational national cancer control policy, strategy, or action plan and the presence of a cancer registry. If those data were present, we looked for additional details on the cancer registry, including whether the registry was population based or hospital based and, if it was population based, whether there was national or subnational coverage. Complete data from Réunion (French), South Sudan, Guadeloupe (French), Martinique (French), Guyana, Puerto Rico, West Bank, Gaza Strip, and New Caledonia (French) were not available and thus were not included in our analyses. The availability of cancer registry data worldwide was depicted as a choropleth map using eSpatial mapping software.^3^

Metadata on income status and health care expenditures were collected from the World Bank.^4^ Countries were classified by income into four categories: high income, upper-middle income, lower-middle income, and low income. We used the χ^2^ test to study the associations between country income status, health care expenditures, the presence of national cancer control policies, and the availability of cancer registry data.

We included 56 high-income countries (HICs), 55 upper-middle-income countries (UMICs), 50 lower-middle-income countries (LMICs), and 29 low-income countries (LICs). In 2015, the global population was 7,316,582,000, of which 1,190,048,000 (16%) lived in HICs, 2,622,663,000 (36%) in UMICs, 2,910,442,000 (40%) in LMICs, and 593,429,000 (8%) in LICs. Although each income group spent a similar percentage of their gross domestic profit on health care (HICs, 7.82%; UMICs, 6.98%; LMICs, 5.86%; and LICs, 6.21%), the mean per capita health expenditure varied widely with HICs spending $3,224, UMICs spending $515, LMICs spending $141, and LICs spending $39 per person (all in US dollars; Table 1).

**Table 1:**
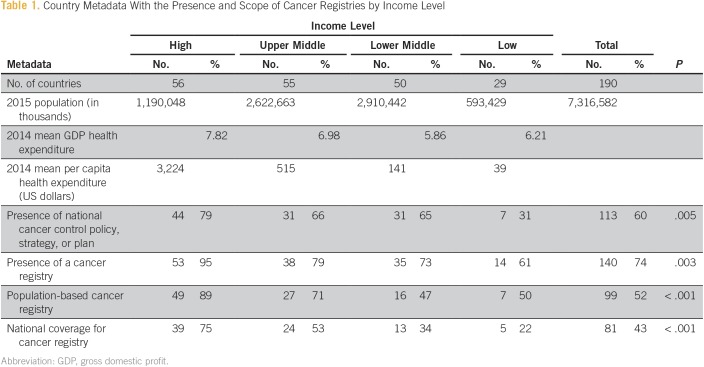
Country Metadata With the Presence and Scope of Cancer Registries by Income Level

**Fig 1:**
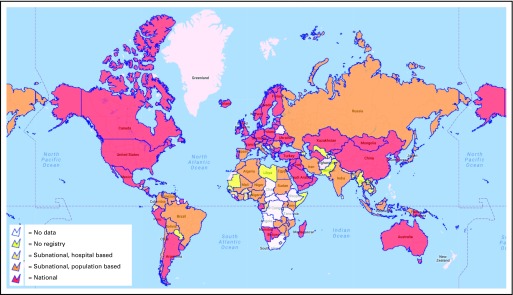
Global variation in the availability of cancer registry data.

Only 60% of all countries had national cancer control policies, strategies, or action plans. Most of these (65%) were HICs or UMICs. Of the 190 countries investigated, 50 (26%) did not have any kind of cancer registry. Of the 140 countries with a registry, 99 (71%) had a population-based registry, and 81 (58%) had national coverage (Fig 1).

Income status was associated with the presence of a registry (*P* = .003) and with the presence of a national cancer control policy (*P* = .005). Only 22% of LICs had a national cancer registry compared with 75% of HICs. Similarly, only 31% of LICs had a national cancer control plan compared with 79% of HICs (Table 1). The presence of a national cancer control policy was also associated with the presence of a cancer registry (*P* < .001). A cancer registry was present in 89% of countries with a national cancer control policy in place compared with 60% of countries without a national policy. This difference held even for LICs in which 72% of those with a national cancer strategy had a cancer registry versus 53% of those without a national strategy.

We observed a large variation in the presence of cancer registry data among countries. The availability of such data was related to a county’s income status and the mean per capita health expenditures. LICs and LMICs were found to have the least cancer registry data. This lack of data has a significant impact on the ability of LMICs to provide cancer care. Without epidemiologic data from population-based registries, assessment of the burden of cancer in a country is limited. This makes it difficult for governments to allocate appropriate resources for cancer care or to measure the performance of specific interventions.

We found an association between the availability of registry data and the presence of a national cancer control policy, even in LMICs. Working with governments to establish national cancer strategies and action plans may be a successful means of encouraging them to create cancer registries. There are several examples of resource-poor countries with viable cancer registries.^5^ We must learn from these examples to lobby clinicians, scientists, administrators, and policy makers to enhance the formation and maintenance of cancer registries globally. Improving global cancer registry data is a vital first step in improving cancer care and cancer research around the world.
